# PKA-CREB-BDNF signaling regulated long lasting antidepressant activities of Yueju but not ketamine

**DOI:** 10.1038/srep26331

**Published:** 2016-05-20

**Authors:** Wenda Xue, Wei Wang, Tong Gong, Hailou Zhang, Weiwei Tao, Lihong Xue, Yan Sun, Fushun Wang, Gang Chen

**Affiliations:** 1 Center for Translational Systems Biology and Neuroscience, Key Laboratory of Integrative Medicine for Brain Diseases, Nanjing University of Chinese Medicine, Nanjing 210023, China; 2 School of Psychology, Nanjing University of Chinese Medicine, Nanjing 210023, China

## Abstract

Yueju confers antidepressant effects in a rapid and long-lasting manner, similar to ketamine. CREB (cAMP-response element binding protein) signaling is implicated in depression pathology and antidepressant responses. However, the role of CREB and associated brain derived neurotrophic factor (BDNF) signaling in rapid and long-lasting antidepressant effects remains unclear. Here, we demonstrated that ICR and Kunming strain mice conferred antidepressant responses lasting for 1 and 5 days, respectively, following a single dose of Yueju. One day post Yueju in Kunming but not ICR strain mice, expression of total and phosphorylated CREB, as well as the CREB signaling activator, PKA (protein kinase A) was up-regulated in the hippocampus. Although BDNF gene expression increased at 3 hours in both strains, it remained up-regulated at 1 day only in Kunming mice. Ketamine showed similar strain-dependent behavioral effects. However, blockade of PKA/CREB signaling blunted the antidepressant effects and reversed the up-regulation of BDNF gene expression by Yueju, but not ketamine. Conversely, blockade of mammalian target of rapamycin signaling led to opposite effects. Taken altogether, prolonged transcriptional up-regulation of hippocampal BDNF may account for the stain-dependent enduring antidepressant responses to Yueju and ketamine, but it was mediated via PKA/CREB pathway only for Yueju.

Depression is among the leading causes of disability worldwide and places a significant emotional and economic burden on patients and their families^1^. Selective serotonin reuptake inhibitors (SSRIs) represent first line antidepressants that are effective for approximately two-thirds of depressed patients^2^. SSRIs typically require 3–6 weeks of chronic treatment before antidepressant effects are observed. Recently, a single dose of ketamine, a noncompetitive N-methyl-D-aspartate receptor (NMDA) antagonist, has been found to alleviate depression symptoms. The effect of a single dose of ketamine is rapid and can last for several days in both humans and animal models^3^,^4^,^5^. Fast-acting antidepressants may be used to treat depression in patients who are not responsive to conventional SSRIs and to rapidly control the suicide ideation^6^,^7^.

As ketamine has toxic and abuse potential, efforts have been made to develop other novel rapid antidepressants. A handful of agents have been identified to display rapid antidepressant-like efficacy, including Yueju, a Chinese herbal medicine which has been used to treat mood disorders for hundreds years^8^,^9^. Like ketamine, a single dose of Yueju quickly increases hippocampal brain derived neurotrophic factor (BDNF) expression in the ICR strain mice. This up-regulated BDNF, non-transcriptional by nature, is crucial for induction of rapid-onset antidepressant activity^5^. One day after ketamine administration, ICR strain mice still show antidepressant response, whereas hippocampal BDNF expression is no longer up-regulated. The antidepressant response does not continue in the following day. Although the dependence of ketamine and Yueju on BDNF for induction of the immediate antidepressant response has been demonstrated, its role in control of the persistent antidepressant response remains elusive.

BDNF expression is regulated by multiple signaling pathways, including cAMP-response element binding (CREB), one of the best studied transcription factors implicated in depression and antidepressant-like responses. Human post-mortem studies reveal lowered CREB level in the hippocampus in major depression and suicide patients^10^. Studies using chronic stress models in rodents show reduced CREB activity^11^,^12^,^13^. Furthermore, chronic but not acute SSRI administration increases CREB activity and its upstream activator PKA^14^,^15^. Modulation of CREB and its target genes results in cellular adaptations underlying the antidepressant effects^16^,^17^,^18^. Chronic treatment with fluoxetine, a SSRI, enhances cAMP levels, subsequently activates PKA and up-regulates CREB mRNA in the hippocampus, cortex and hypothalamus of the chronically stressed rats^19^. Conversely, blockade of CREB signaling blunts the antidepressant effects of chronic SSRIs^20^. CREB signaling regulates expression of genes that promote synaptic and neural plasticity, including BDNF, as evidenced by the presence of CRE elements in the promoter region of BDNF^21^. CREB-BDNF signaling has been suggested to be critical in numerous neuronal biological processes, including cell survival, synaptic structure, and synaptic plasticity^22^,^23^. A prolonged activation of CREB-BDNF signaling may serve to promote the persistent antidepressant effects of Yueju or ketamine. However, this has not been examined in depth.

Previous studies showed that an antidepressant response in the tail suspension test lasted for only 1 day in ICR strain mice^8^, whereas it lasted for 5 days in Kunming (KM) strain mice exposed to chronic stress^24^. We hypothesize that the difference in antidepressant action between strains is due to a difference in the time course of CREB-BDNF signaling in the two strains. Here, we first examined the strain difference in the duration of the antidepressant response of Yueju. The expression patterns of CREB and BDNF at different time points post Yueju were assessed in ICR and KM strains. Additionally, we investigated the role of the activation of CREB upstream regulator PKA, and tested whether blockade of the PKA-CREB signaling influenced BDNF expression as well as the antidepressant effects of Yueju. Finally, as the persistent antidepressant activity of ketamine was suggested to require activation of mammalian target of rapamycin (mTOR) signaling, which is also implicated in the effects of Yueju^24^, we thus tested whether BDNF gene expression and antidepressant activity of Yueju was dependent on mTOR signaling.

## Results

### Yueju conferred a longer duration of antidepressant response in KM compared to ICR mice

**Figure 1:**
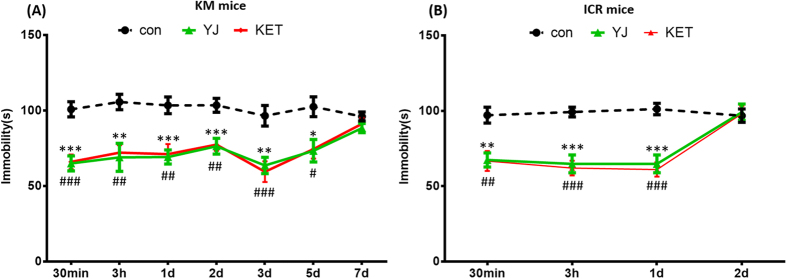
Time course of Yueju (YJ) and ketamine (KET) mediated antidepressant-like behavioral effects. Independent groups of mice were used at each time point and for each drug treatment, to avoid behavioral habituation. (**A**) Antidepressant activities in KM mice. Analysis of variance (ANOVA) *F*_(2,20)_ = 60.161, *p* < 0.0001 for treatment; *F*_(6,210)_ = 3.374, *p* < 0.01 for duration of response; *F*_(12,231)_ = 1.188, *p* = 0.293 for treatment-duration interaction. Therefore, we examined treatment effects by time point. n = 11/group. (**B**) Antidepressant activities in ICR mice. Analysis of variance (ANOVA) *F*_(2,11)_ = 33.893, *p* < 0.0001 for treatment; *F*_(3,108)_ = 14.033, *p* < 0.0001 for duration of response; *F*_(6,120)_ = 4.471, *p* < 0.001 for treatment-duration interaction. n = 10/group. *p < 0.05, **p < 0.01, ***p < 0.001, YJ compared to control; ^#^*p* < 0.05, ^##^*p* < 0.01, ^###^*p* < 0.001, KET compared to control. Data represent means ± SEM.

The time course of depression-like behavior following a single dose of Yueju or ketamine in KM (Fig. 1A) and ICR (Fig. 1B) mice is illustrated. To avoid potential confounding effects of repeated testing, individual animals were tested once. In both strains, there were significant decreases in the time spent immobile at 30 minutes (*F*_(2,32)_ = 17.061, *p* < 0.05), 3 hours (*F*_(2,32)_ = 9.065, *p* < 0.05), 1 day (*F*_(2,32)_ = 11.551, *p* < 0.05), 2 days (*F*_(2,32)_ = 10.713, *p* < 0.05), 3 days (*F*_(2,32)_ = 10.152, *p* < 0.05), and 5 days (*F*_(2,32)_ = 5.89, *p* < 0.05) post Yueju or ketamine administration in KM mice (Fig. 1A). There were also significant decreases in the time spent immobile at 30 min (*F*_(2,29)_ = 9.67, *p* < 0.05), 3 hours (*F*_(2,29)_ = 18.652, *p* < 0.05), and 1 day (*F*_(2,29)_ = 20.607, *p* < 0.05) post Yueju or ketamine administration in ICR mice (Fig. 1B). The antidepressant-like effect was no longer apparent in KM mice at 7 days (*F*_(2,32)_ = 1.353, *p* = 0.274), or in ICR mice at 2 days (*F*_(2,29)_ = 0.054, *p* = 0.948) post Yueju administration. This pattern was also evident in ketamine-treated animals. In the open field test, neither Yueju nor ketamine (30 min post-drug) altered the total distance traveled (*F*_(2, 32)_ = 0.352, *p* = 0.706) or the total time spent in center (*F*_(2, 32)_ = 0.036, *p* = 0.965) in KM mice, similar to ICR mice^8^.

### Yueju treatment induced a long-lasting up-regulation of BDNF expression in KM mice

**Figure 2:**
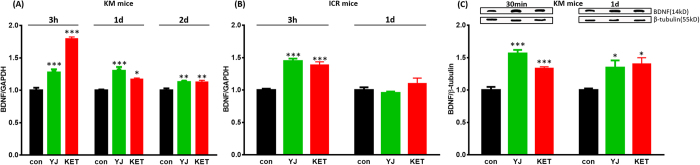
BDNF mRNA in KM or ICR mice as well as protein expression in KM mice after an acute dose of Yueju (YJ) and Ketamine (KET). (**A**) BDNF mRNA expression significantly increased in KM mice at 3 hours (*F*_(2,17)_ = 108.764, *p* < 0.05), 1 day (*F*_(2,17)_ = 17.708, *p* < 0.05) and 2 days (*F*_(2,17)_ = 9.499, *p* < 0.05) after Yueju and ketamine administration. n = 6/group. (**B**) BDNF mRNA expression increased in ICR mice at 3 hours (*F*_(2,17)_ = 43.605, *p* < 0.05) but not at 1 day (*F*_(2,17)_ = 1.779, *p* = 0.203) after Yueju or ketamine administration. n = 6/group. (**C**) BDNF protein expression in KM mice 30 minutes (*p* < 0.01) or 1 day (*p* < 0.05) post Yueju and ketamine. n = 5/group. *p < 0.05, **p < 0.01, ***p < 0.001, compared to control. Data represent means ± SEM.

BDNF mRNA expression did not alter 30 minutes post-Yueju in KM strain mice (*F*_(2,17)_ = 1.493, *p* = 0.256). However, there was a significant increase in BDNF mRNA levels at 3 hours (*F*_(2,17)_ = 108.764, *p* < 0.05), 1 day (*F*_(2,17)_ = 17.708, *p* < 0.05), and 2 days (*F*_(2,17)_ = 9.499, *p* < 0.05) after Yueju and ketamine treatment in KM mice (Fig. 2A). In contrast, BDNF mRNA increased only at 3 hours (*F*_(2,17)_ = 43.605, *p* < 0.05), but not 1 day (*F*_(2,17)_ = 1.779, *p* = 0.203) post Yueju or ketamine in ICR mice (Fig. 2B). There was a significant increase in BDNF protein expression in KM mice 1 day (*F*_(2,17)_ = 6.568, *p* < 0.05) after Yueju (Fig. 2C). Despite the lack of change at a transcriptional level, BDNF protein expression significantly increased 30 min after Yueju treatment in KM mice (*F*_(2,17)_ = 41.119, *p* < 0.05, Fig. 2C), similar to ICR strain mice^8^. Acute ketamine showed a similar pattern of BDNF mRNA and protein expression in both strains (Fig. 2).

### Yueju induced long-term up-regulation of CREB mRNA and protein expression in KM mice

**Figure 3:**
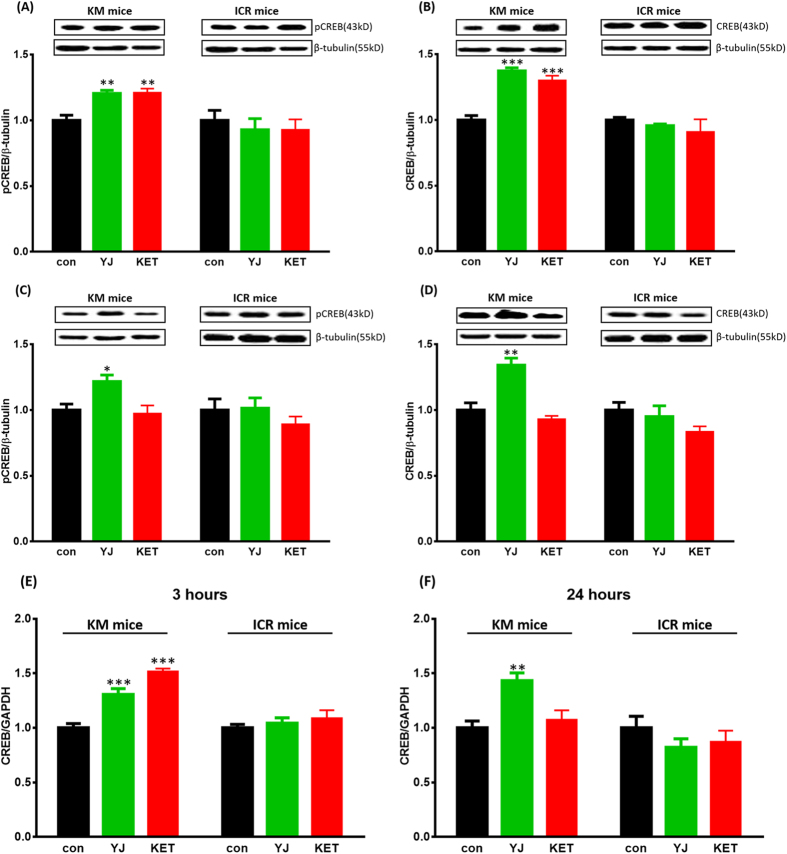
CREB mRNA and protein expression after an acute dose of Yueju (YJ) and Ketamine (KET) in the hippocampus of KM or ICR mice. pCREB (**A**) and total CREB (**B**) protein expression in KM and ICR mice 1 day after YJ and KET treatment. pCREB (**C**) and total CREB (**D**) protein expression in KM mice and ICR mice 2 days after YJ and KET treatment. CREB mRNA expression in KM and ICR mice 3 hours (**E**) and 1 day (**F**) after Yueju treatment. *p < 0.05, **p < 0.01, ***p < 0.001, compared to control, n = 4~6. Data represent means ± SEM.

There were strain dependent differences in total CREB and pCREB protein expression in the hippocampus 1 day and 2 days after Yueju and ketamine administration. In ICR mice, pCREB, total CREB protein expression and the ratio of pCREB/CREB was unchanged 1 day (Fig. 3A,B) or 2 days (Fig. 3C,D) after Yueju or ketamine administration. In KM mice, pCREB significantly increased 1 day after Yueju and ketamine (*F*_(2,11)_ = 14.050, *p* < 0.05, Fig. 3A). Additionally, total CREB expression significantly increased 1 day after Yueju and ketamine (*F*_(2,11)_ = 39.725, *p* < 0.05, Fig. 3B), whereas the ratio of phosphorylated CREB to total CREB (pCREB/CREB) was unchanged by Yueju or ketamine (*F*_(2,11)_ = 0.943, *p* = 0.425). In the following day, only Yueju, but not ketamine, continued to significantly increase the expression of pCREB (*F*_(2,11)_ = 6.560, *p* < 0.05, Fig. 3C) and total CREB (*F*_(2,11)_ = 22.774, *p* < 0.05, Fig. 3D) in KM mice. These findings suggest that the increased pCREB expression is contributed by increased expression of total CREB.

The increase in CREB protein expression may be due to transcriptional up-regulation, and thus we also assessed CREB gene expression in the hippocampus at 30 minutes (*F*_(2,17)_ = 0.103, *p* = 0.903), 3 hours (*F*_(2,17)_ = 0.652, *p* = 0.535) and 1 day (*F*_(2,17)_ = 0.920, *p* = 0.420) post-drug in ICR mice. There was no change in CREB mRNA expression at any time point measured post-Yueju or -ketamine in ICR mice. Although neither Yueju nor ketamine altered CREB gene expression in KM mice at 30 min (*F*_(2,17)_ = 0.303, *p* = 0.743), there was significant increase 3 hours (*F*_(2,17)_ = 39.936, *p* < 0.05, Fig. 3E) after Yueju or ketamine in KM mice. Additionally, only Yueju (*p* < 0.01), but not ketamine, continued to increase CREB mRNA expression 1 day after administration (*F*_(2,17)_ = 9.862, *p* < 0.05, Fig. 3F).

### Blockade of PKA-CREB signaling blunted antidepressant effects and up-regulation of BDNF gene expression by Yueju, but not ketamine

**Figure 4:**
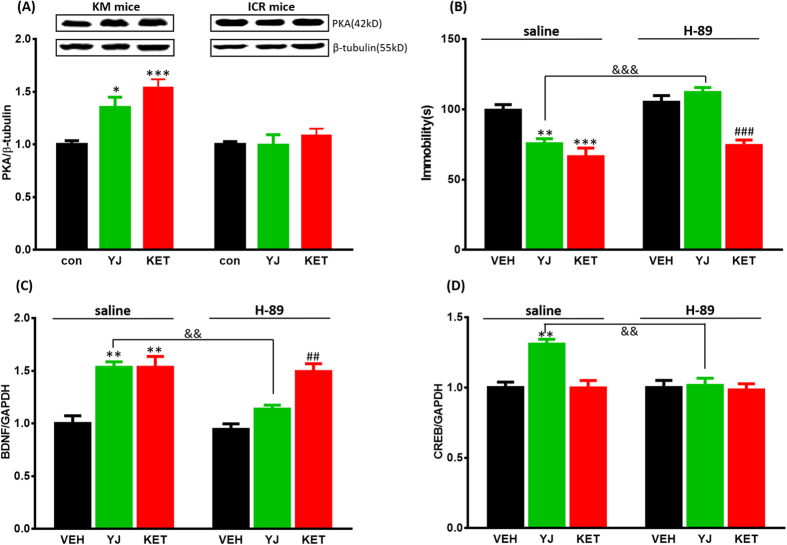
PKA protein expression and the effect a PKA antagonist on antidepressant response and BDNF/CREB gene expression in the hippocampus after a single Yueju/ketamine treatment. (**A**) PKA protein expression 1 day after Yueju (YJ) and Ketamine (KET) treatment in KM and ICR mice. PKA protein significantly increased in KM mice (*F*_(2,17)_ = 13.228, *p* < 0.001) but not in ICR mice (*F*_(2,17)_ = 0.541, *p* = 0.593). n = 6/group. *p < 0.05, ***p < 0.001, compared to control. (**B**) The effect of saline (*F*_(2,23)_ = 13.413, *p* < 0.001) or H-89 (*F*_(2,23)_ = 24.307, *p* < 0.001) pretreatment on immobility time in the tail suspension test 1 day after Yueju and ketamine treatment in KM mice. n = 8/group. **p < 0.01, ***p < 0.001, compared to vehicle (VEH, saline); ^###^*p* < 0.001, compared to vehicle (H-89); ^&&&^*p* < 0.001, compared to YJ. (**C**) The effect of saline (*F*_(2,11)_ = 15.173, *p* < 0.01) or H-89 (*F*_(2,11)_ = 9.746, *p* < 0.01) pretreatment on BDNF mRNA expression 1 day after Yueju and ketamine treatment in KM mice. n = 4/group. **p < 0.01, compared to vehicle (saline); ^##^*p* < 0.01, compared to vehicle (H-89); ^&&^*p* < 0.01, compared to YJ. (**D**) The effect of saline (*F*_(2,11)_ = 11.688, *p* < 0.01) or H-89 (*F*_(2,11)_ = 0.033, *p* = 0.968) pretreatment on CREB mRNA expression 1 day after Yueju and ketamine treatment in KM mice. n = 4/group. **p < 0.01, compared to vehicle (saline); ^&&^*p* < 0.01, compared to YJ. Data represent means ± SEM.

Consistent with CREB expression, PKA protein expression was up-regulated 1 day post Yueju and ketamine (*F*_(2,17)_ = 13.228, *p* < 0.001) treatment in KM mice, but not in ICR mice (*F*_(2,17)_ = 0.541, *p* = 0.593, Fig. 4A).

To further evaluate the role of PKA/CREB/BDNF signaling in antidepressant actions of Yueju and ketamine, animals were pretreated with a PKA inhibitor H-89. The pre-treatment of KM mice with H-89 was able to reverse the antidepressant-like effect of Yueju. A two-way ANOVA revealed significant differences for the H-89 pre-treatment (*F*_(1,28)_ = 28.439, *p* < 0.001), Yueju treatment (*F*_(1,28)_ = 4.636, *p* < 0.05) and H-89 × Yueju interaction (*F*_(1,28)_ = 15.186, *p* < 0.001, Fig. 4B). The administration of H-89 alone did not affect the immobility in the tail suspension test in non-Yueju mice (*p* = 0.369), but significantly increased it in the Yueju-treated mice (p < 0.01). In contrast, H-89 couldn’t block the antidepressant-like effect of ketamine: a two-way ANOVA revealed the significant main effect of ketamine treatment (*F*_(1,28)_ = 45.366, *p* < 0.001), but not H-89 pre-treatment (*F*_(1,28)_ = 2.064, *p* = 0.162) or H-89 × ketamine interaction (*F*_(1,28)_ = 0.06, *p* = 0.808).

Consistent with blocking the behavioral effects of Yueju, H-89 pretreatment also reversed up-regulation of BDNF gene expression induced by Yueju. A two-way ANOVA revealed significant differences for the H-89 pre-treatment (*F*_(1,12)_ = 24.961, *p* < 0.001), Yueju treatment (*F*_(1,12)_ = 32.005, *p* < 0.001), and H-89 × Yueju interaction (*F*_(1,12)_ = 15.892, *p* < 0.01, Fig. 4C). The administration of H-89 alone did not affect the BDNF mRNA level in non-Yueju mice (*p* = 0.558), but significantly decreased it in the Yueju-treated mice (*p* < 0.01). In contrast, H-89 couldn’t block the increase of BDNF mRNA induced by ketamine: a two-way ANOVA revealed the significant main effect of ketamine treatment (*F*_(1,12)_ = 48.365, *p* < 0.001), but not H-89 pre-treatment (*F*_(1,12)_ = 0.38, *p* = 0.549) or H-89 × ketamine interaction (*F*_(1,12)_ = 0.01, *p* = 0.921).

H-89 pretreatment also reversed up-regulation of CREB gene expression induced by Yueju. A two-way ANOVA revealed significant differences for the H-89 pre-treatment (*F*_(1,12)_ = 13.041, *p* < 0.01), Yueju treatment (*F*_(1,12)_ = 8.871, *p* < 0.05) and H-89 × Yueju interaction (*F*_(1,12)_ = 9.936, *p* < 0.01, Fig. 4D). The administration of H-89 alone did not affect the CREB mRNA level in non-Yueju mice (*p* = 0.752), but significantly decreased it in the Yueju-treated mice (*p* < 0.01). Meanwhile, neither ketamine nor H-89 affected the CREB mRNA level (two way ANOVA, *F*_(3,15)_ = 0.053, *p* = 0.983). These results suggest that the antidepressant effects of Yueju, but not ketamine, are dependent on the PKA/CREB/BDNF pathway.

### Blockade of mTOR signaling blunted antidepressant effects and up-regulation of BDNF gene expression by ketamine, but not Yueju

**Figure 5:**
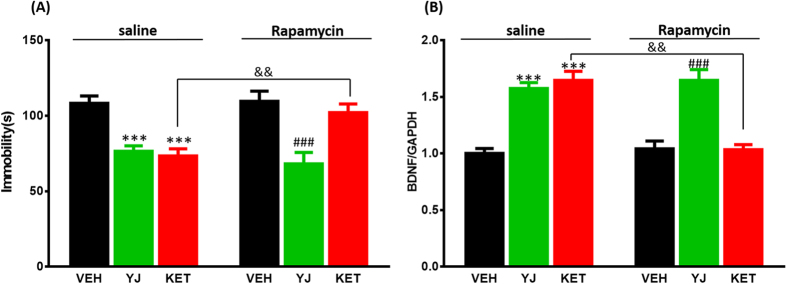
Effects of mTOR blockade with rapamycin pretreatment on immobility times in the tail suspension test in KM mice and BDNF gene expression 1 day post-Yueju and ketamine. (**A**) The effect of saline (*F*_(2,23)_ = 18.818, *p* < 0.001) or rapamycin (*F*_(2,23)_ = 10.734, *p* < 0.001) pretreatment on immobility time in the tail suspension test 1 day after Yueju and ketamine treatment in KM mice. n = 8/group. ***p < 0.001, compared to vehicle (VEH, saline); ^###^*p* < 0.001, compared to vehicle (rapamycin); ^&&^*p* < 0.01, compared to KET. (**B**) The effect of saline (*F*_(2,11)_ = 34.595, *p* < 0.001) or rapamycin (*F*_(2,11)_ = 23.702, *p* < 0.001) pretreatment on BDNF mRNA expression 1 day after Yueju and ketamine treatment in KM mice. n = 4/group. ***p < 0.001, compared to vehicle (saline); ^###^*p* < 0.001, compared to vehicle (rapamycin); ^&&^*p* < 0.01, compared to KET. Data represent means ± SEM.

Additionally, we assessed the role of mTOR signaling in the antidepressant effects of Yueju or ketamine. Animals were pretreated with an mTOR inhibitor rapamycin before administration of Yueju and ketamine. The pre-treatment of KM mice with rapamycin was able to reverse the antidepressant-like effect of ketamine: a two-way ANOVA revealed significant main effect of rapamycin pre-treatment (*F*_(1,28)_ = 7.243, *p* < 0.05), ketamine treatment (*F*_(1,28)_ = 14.328, *p* < 0.001) and rapamycin × ketamine interaction (*F*_(1,28)_ = 6.003, *p* < 0.05, Fig. 5A). The administration of rapamycin alone did not affect the immobility in the tail suspension test in non-ketamine mice (*p* = 0.874), but significantly increased it in the ketamine-treated mice (*p* < 0.01). In contrast, rapamycin couldn’t block the antidepressant-like effect of Yueju: a two-way ANOVA revealed the significant main effect of Yueju treatment (*F*_(1,28)_ = 38.210, *p* < 0.05), but not rapamycin pre-treatment (*F*_(1,28)_ = 0.349, *p* = 0.56) or rapamycin × Yueju interaction (*F*_(1,28)_ = 0.67, *p* = 0.42).

Consistent with blocking the behavioral effects of ketamine, rapamycin pretreatment also reversed up-regulation of BDNF gene expression induced by ketamine. A two-way ANOVA revealed the significant differences for rapamycin pre-treatment (*F*_(1,12)_ = 22.113, *p* < 0.001), ketamine treatment (*F*_(1,12)_ = 27.588, *p* < 0.001) and rapamycin × ketamine interaction (*F*_(1,12)_ = 29.005, *p* < 0.01, Fig. 5B). The administration of rapamycin alone did not affect the BDNF mRNA level in non-ketamine mice (*p* = 0.558), but significantly decreased it in the ketamine-treated mice (*p* < 0.01). In contrast, rapamycin couldn’t block the increase in BDNF mRNA induced by Yueju: a two-way ANOVA revealed the main effect of Yueju treatment (*F*_(1,12)_ = 76.054, *p* < 0.001), but not rapamycin pre-treatment (*F*_(1,12)_ = 0.703, *p* = 0.418) or rapamycin × Yueju interaction (*F*_(1,12)_ = 0.05, *p* = 0.826). These results suggest that the antidepressant effect and increased BDNF gene expression by ketamine, but not Yueju, is dependent on mTOR pathway.

## Discussion

The present study aimed to dissect molecular and neurobiological mechanisms responsible for long-lasting antidepressant responses after Yueju. Antidepressant effects of Yueju or ketamine last for 1 or 5 days in ICR and KM mice, respectively. One day after Yueju and ketamine treatment, there was increased expression of total and phosphorylated CREB protein in KM but not ICR mice. Importantly, the CREB upstream regulator, PKA, and the downstream effector, BDNF, also showed a similar strain-dependent expression pattern. Although PKA/CREB/BDNF expression were up-regulated 1 day post administration of either ketamine or Yueju, inhibition of PKA-CREB signaling only reversed BDNF gene expression and the antidepressant effect of Yueju, but not ketamine, indicating that the PKA/CREB/BDNF pathway was required for the lasting antidepressant effects of Yueju but not ketamine.

CREB signaling in the hippocampus has been implicated in affective and cognitive behaviors, and previous studies have shown strain dependent differences in CREB signaling that may contribute to differential learning and memory related behaviors^25^,^26^,^27^,^28^,^29^. CREB activation is a hallmark of the neural plasticity responsible for antidepressant effects after chronic SSRI administration^30^. Here we demonstrated the strain-dependent differences in CREB signaling linked to a persistent antidepressant response after a single dose of Yueju. pCREB signaling was up-regulated 1 day post-Yueju and sustained at least for one more day in KM mice, contrasting to no change in CREB signaling in ICR mice, which paralleled the longer duration of antidepressant response in KM mice. The increased pCREB expression is partly attributable to increased gene and protein expression of total CREB as well as increased activation of PKA. It is notable that CREB signaling in the hippocampus was not up-regulated by 30 minutes post Yueju, whereas both strains showed antidepressant effects, indicating that CREB signaling was not responsible for their immediate antidepressant responses. Importantly, up-regulated expression of CREB was detected 3 hours post Yueju, with increased CREB and BDNF mRNA expression in KM mice. Inhibition of CREB signaling abolished up-regulation of BDNF gene expression and antidepressant response at 1 day post Yueju, supporting the hypothesis that CREB-BDNF signaling is required for the maintenance of antidepressant response to Yueju.

Although Yueju and ketamine both activated the PKA/CREB pathway, our further studies showed the role of the pathway was different. In Yueju-treated mice, up-regulation of BDNF mRNA expression was temporally aligned with increased total and phosphorylated CREB expression, and blockade of PKA/CREB signaling reversed the up-regulation of BDNF expression as well as the antidepressant effect of Yueju. In contrast, after treatment with ketamine, the increase in CREB expression was only transitory whereas the BDNF gene expression continued to be up-regulated, suggesting the two events may be independent. Furthermore, blockade of PKA/CREB signaling failed to change BDNF expression or antidepressant effect of ketamine. These findings suggest that CREB signaling does not play a primary role in antidepressant actions of ketamine, unlike Yueju. This finding is agreement with reports that hippocampal CREB and PKA protein expression is not associated with antidepressant response of ketamine^31^. Conversely, we found that the mTOR inhibitor rapamycin could reverse the antidepressant effect of ketamine, but not Yueju, supporting the hypothesis that long-lasting antidepressant action of ketamine may rely mainly on mTOR-associated signaling^4^. As Yueju is able to reverse the deficient mTOR-related signaling in chronically stressed animals^24^, there may exist a cross-talk between PKA/CREB and mTOR signaling, which warrants further investigation.

The present study demonstrates for the first time that up-regulation of BDNF expression may be universally involved in the persistent antidepressant response of Yueju and ketamine in both KM and ICR mouse strains. This is in contrast to the specific role of CREB in the persistent antidepressant response to Yueju. It has been well documented that chronic SSRI treatment activates transcription factors, including CREB, leading to an increase in the expression of neurotrophic factors, including BDNF, and their receptors^30^,^32^. For ketamine, the requirement of BDNF for initiation of the antidepressant response has been demonstrated previously^5^. Our studies showed that, without changes in CREB activation or BDNF mRNA expression, BDNF protein in the hippocampus is up-regulated quickly after ketamine or Yueju treatment in both KM and ICR mice. This non-transcriptional BDNF up-regulation was thus responsible for the antidepressant response immediately post Yueju or ketamine administration. This is followed by up-regulation of BDNF mRNA expression, likely via CREB signaling for Yueju and mTOR signaling for ketamine. ICR mice with a shorter time course of BDNF mRNA expression displayed a shorter antidepressant time course than KM mice. The transcriptional up-regulation of BDNF may regulate the cellular signaling governing the persistent antidepressant responses, as reversal of BDNF gene expression by blocking either CREB or mTOR signaling was associated with a blunted antidepressant response 1 day post Yueju or ketamine administration, respectively. Although the molecular link from the initial non-transcriptional BDNF protein upregulation to the following transcriptional BDNF up-regulation remains to be determined, it has been shown that BDNF, via activating its receptor TrkB, can induce the activation of mTOR signaling and CREB signaling^4^,^33^. Furthermore, a recent study demonstrates that in the cultured neurons, BDNF can initiate self-amplification of BDNF mRNA expression via multiple signaling pathways including PKA-CREB pathway^34^. Therefore, the initial non-transcriptional up-regulation of BDNF may still be of importance in inducing the cellular signaling that promotes BDNF gene expression for long-lasting antidepressant responses. This can be elucidated by manipulating BDNF gene expression in a time-dependent manner.

The present study identified for the first time strain-dependent PKA/CREB/BDNF signaling that regulates long-lasting antidepressant responses to Yueju. Our results also indicate that continuous up-regulation of hippocampal BDNF mRNA is essential for the persistent antidepressant response, either for Yueju or ketamine. Further studies should address the genetic variants and gene networks that perturb BDNF and other signaling commonly responsible for individual differences in antidepressant responses after ketamine or Yueju treatment. As long-lasting antidepressant efficacy is implicated in sustained therapeutic outcomes, elucidation of the underlying mechanisms and genetic variants influencing the responses may shed new light on effective personalized medicines for depression.

## Materials and Methods

### Subjects

Male Kunming (KM) and ICR (25 ~ 30 g), outbred strains of mice, aged 7–8 w, were purchased from Academy of Military Medical Sciences. KM, the most commonly used outbred mouse strain line in China, originated from the Swiss mice from the Indian Haffkine Institute in 1944. Abundant genetic variations of KM mice have been characterized^35^,^36^. Mice were kept in an air-conditioned (22–25 °C) room with a 12 h light/dark cycle with free access to food and water. Only male mice were used to avoid the potential gender difference in antidepressant responses^37^,^38^. All animal procedures were carried out in accordance with the Guide for the Care and Use of Laboratory Animals approved by the Institutional Animal Care and Use Committee at Nanjing University of Chinese medicine.

### Drugs and treatment

Yueju was processed and purified as described in Xue *et al*.^8^. Briefly, the medicinal plants used to prepare Yueju are *Cyperusrotundus L.* (CR), *Ligusticum chuanxiong Hort.* (LC), *Gardenia jasminoides Ellis.* (GJ), *Atractylodeslancea(Thunb.)DC.* (AL) and *Massa Fermentata* (MF). All the medicinal plants were purchased from Nanjing GuoYi Clinical, Medicinal Material Department (Nanjing, China). The herbal mixture was powdered, immersed in 95% of ethanol with constant shaking and filtered. This procedure was repeated three times, and the collected solvent was evaporated at low pressure and medium temperature (<55 °C) until ethanol was completely eliminated. The extract of Yueju was dispersed in Tween 80 solution (0.5%, w/v in saline) and administrated intragastrically (270 mg/ml, i.g.). Quality control of the preparation was performed as described previously using HPLC fingerprint analysis. Different samples of Yueju preparation were revealed very similar and suitable^8^. The doses of ketamine (50 mg/kg in ICR mice^8^ and 30 mg/kg in KM mice^24^) and Yueju^8^,^24^ were optimized based on a pilot assessment of a dose-response relationship in the strain of mice as we described in previous studies. 0.5%, w/v Tween 80 in saline solution via i.g. and saline via i.p. served as the vehicle controls, and their behavioral data or western blot were collapsed as there were no statistical differences between them. H-89 at 10 mg/kg (Sigma, St. Louis, MO, USA) and rapamycin at 5 mg/kg (Sigma, St. Louis, MO, USA), were dissolved in 0.5% DMSO (dimethyl sulfoxide) and distilled water, respectively, and were injected i.p. 30 minutes before ketamine, Yueju, or vehicle administration.

### Tail suspension test (TST)

Mice were assessed in the TST, which was performed with a computerized device that allowed four animals to be tested at one time. In a chamber that was acoustically and visually isolated, an individual mouse was suspended 50 cm above the floor by adhesive tape placed approximately 1 cm from the tip of the tail. The activities of the animal were videotaped. ANY-maze software (Stoeling Co.Ltd., USA) was used to calculate the total time spent immobile during the last 4 min in a 6-min testing period^39^.

### Open field Test (OFT)

The OFT assesses locomotor activity and anxiety-like behavior in a bright-lit open area. Testing was performed for 5 min in a well-illuminated (∼300 lux) transparent acrylic cage (40 × 40 × 15 cm). The mice were gently placed on the center and left to explore the area for 5 min. The digitized image of the path taken by each mouse was tracked by camera, and the total running distances (locomotor activity) and spending times in center were analyzed using ANY-maze software. The testing apparatus was thoroughly cleaned with 70% ethanol and then dried between each animal.

### Western blots

Mice were sacrificed by decapitation at the designated time points. The hippocampus was dissected out and put into ice cold tubes containing an enzyme inhibitor. Brain tissue was homogenized and western blot analysis was carried out, using primary antibodies for rabbit pCREB (1:500), rabbit CREB (1:1000), rabbit PKAC-α (1:1000) and rabbit β-tubulin (1: 5000) (all from Cell Signal Inc., CA, USA), BDNF (1:200, Santa Cruz). A secondary antibody (1:2000) conjugated with horseradish peroxidase was used. Immunoreactivity was visualized by ECL reagent. Blots were visualized using the SuperSignal West Pico Chemiluminescent Substrate (Thermo Fisher Scientific Inc.) and were shown as density relative to β-tubulin. All experiments were performed 3 times.

### Reverse transcription quantitative real-time PCR (RT-qPCR)

RNA was isolated from the whole hippocampus (ventral and dorsal) using Trizol reagent (Invitrogen) and were reverse transcribed to cDNA using the SYBR PrimeScript RT-PCR Kit (Takara). RT-qPCR was performed using 1.5 *μ*L of cDNA and the SYBR Green Master Mix reagent (Takara). The following primers were used: BDNF forward, 5′-CCA TAAAGG ACG CGG ACT TGT ACA-3′; BDNF reverse, 5′-AGACAT GTT TGC GGC ATC CAG-3′; CREB forward, 5′-TCAGCCGGGTACTACCATTC-3′; CREB reverse, 5′-TCTCTTGCTGCTTCCCTGTT-3′; GAPDH forward, 5′-AAC GAC CCC TTC ATT GAC-3′; and GAPDH reverse, 5′-TCC ACG ACA TAC TCA GCA C-3′. The fold-change in BDNF or CREB expression (coding exon) was normalized to GAPDH. The RT-qPCR was carried out using instructions from the manufacturer’s manual. The primers were tested for quality and specificity by melt-curve analysis, gel electrophoresis and appropriate negative controls. Relative expression values were obtained using the ΔΔCT method.

### Statistical analyses

All data were presented as means ± S.E.M. Differences among groups were determined using one-way ANOVA or two-way ANOVA, followed by a Bonferroni post hoc analysis if appropriate. *p* < 0.05 was the accepted level of significance.

## Additional Information

**How to cite this article**: Xue, W. *et al*. PKA-CREB-BDNF signaling regulated long lasting antidepressant activities of Yueju but not ketamine. *Sci. Rep.* **6**, 26331; doi: 10.1038/srep26331 (2016).

## References

[b1] Wong M. L. & Licinio J. Research and treatment approaches to depression. Nat Rev Neurosci 2, 343–351, doi: 10.1038/35072566 (2001). 11331918

[b2] Trivedi M. H. . Evaluation of outcomes with citalopram for depression using measurement-based care in STAR*D: implications for clinical practice. Am J Psychiatry 163, 28–40, doi: 10.1176/appi.ajp.163.1.28 (2006). 16390886

[b3] Berman R. M. . Antidepressant effects of ketamine in depressed patients. Biological psychiatry 47, 351–354 (2000). 10686270 10.1016/s0006-3223(99)00230-9

[b4] Li N. . mTOR-dependent synapse formation underlies the rapid antidepressant effects of NMDA antagonists. Science 329, 959–964, doi: 10.1126/science.1190287 (2010). 20724638 PMC3116441

[b5] Autry A. E. . NMDA receptor blockade at rest triggers rapid behavioural antidepressant responses. Nature 475, 91–95, doi: 10.1038/nature10130 (2011). 21677641 PMC3172695

[b6] Zarate C. A. Jr. . A randomized trial of an N-methyl-D-aspartate antagonist in treatment-resistant major depression. Arch Gen Psychiatry 63, 856–864, doi: 10.1001/archpsyc.63.8.856 (2006). 16894061

[b7] Ballard E. D. . Assessing measures of suicidal ideation in clinical trials with a rapid-acting antidepressant. Journal of psychiatric research 68, 68–73, doi: 10.1016/j.jpsychires.2015.06.003 (2015). 26228403 PMC4522045

[b8] Xue W. . Yueju pill rapidly induces antidepressant-like effects and acutely enhances BDNF expression in mouse brain. Evidence-based complementary and alternative medicine : eCAM 2013, 184367, doi: 10.1155/2013/184367 (2013). 23710213 PMC3654702

[b9] Wu R. . A role of Yueju in fast-onset antidepressant action on major depressive disorder and serum BDNF expression: a randomly double-blind, fluoxetine-adjunct, placebo-controlled, pilot clinical study. Neuropsychiatric disease and treatment 11, 2013–2021, doi: 10.2147/NDT.S86585 (2015). 26273204 PMC4532216

[b10] Duric V. . A negative regulator of MAP kinase causes depressive behavior. Nat Med 16, 1328–1332, doi: 10.1038/nm.2219 (2010). 20953200 PMC3066515

[b11] Lu J. . Acupuncture Activates ERK-CREB Pathway in Rats Exposed to Chronic Unpredictable Mild Stress. Evidence-based complementary and alternative medicine : eCAM 2013, 469765, doi: 10.1155/2013/469765 (2013). 23843874 PMC3703360

[b12] Laifenfeld D., Karry R., Grauer E., Klein E. & Ben-Shachar D. Antidepressants and prolonged stress in rats modulate CAM-L1, laminin, and pCREB, implicated in neuronal plasticity. Neurobiol Dis 20, 432–441, doi: 10.1016/j.nbd.2005.03.023 (2005). 15905095

[b13] Gronli J. . Chronic mild stress inhibits BDNF protein expression and CREB activation in the dentate gyrus but not in the hippocampus proper. Pharmacol Biochem Behav 85, 842–849, doi: 10.1016/j.pbb.2006.11.021 (2006). 17204313

[b14] Pinnock S. B., Blake A. M., Platt N. J. & Herbert J. The roles of BDNF, pCREB and Wnt3a in the latent period preceding activation of progenitor cell mitosis in the adult dentate gyrus by fluoxetine. PLoS One 5, e13652, doi: 10.1371/journal.pone.0013652 (2010). 21048974 PMC2965105

[b15] Thome J. . cAMP response element-mediated gene transcription is upregulated by chronic antidepressant treatment. The Journal of neuroscience : the official journal of the Society for Neuroscience 20, 4030–4036 (2000). 10818138 10.1523/JNEUROSCI.20-11-04030.2000 PMC6772651

[b16] Duman R. S., Malberg J., Nakagawa S. & D’Sa C. Neuronal plasticity and survival in mood disorders. Biological psychiatry 48, 732–739 (2000). 11063970 10.1016/s0006-3223(00)00935-5

[b17] Shaywitz A. J. & Greenberg M. E. CREB: a stimulus-induced transcription factor activated by a diverse array of extracellular signals. Annu Rev Biochem 68, 821–861, doi: 10.1146/annurev.biochem.68.1.821 (1999). 10872467

[b18] Silva A. J., Kogan J. H., Frankland P. W. & Kida S. CREB and memory. Annu Rev Neurosci 21, 127–148, doi: 10.1146/annurev.neuro.21.1.127 (1998). 9530494

[b19] Li Y. C. . Antidepressant-like effects of curcumin on serotonergic receptor-coupled AC-cAMP pathway in chronic unpredictable mild stress of rats. Progress in neuro-psychopharmacology & biological psychiatry 33, 435–449, doi: 10.1016/j.pnpbp.2009.01.006 (2009). 19302828

[b20] Hu Y. . Hippocampal nitric oxide contributes to sex difference in affective behaviors. Proc Natl Acad Sci USA 109, 14224–14229, doi: 10.1073/pnas.1207461109 (2012). 22891311 PMC3435162

[b21] Finkbeiner S. . CREB: a major mediator of neuronal neurotrophin responses. Neuron 19, 1031–1047 (1997). 9390517 10.1016/s0896-6273(00)80395-5

[b22] Chen B., Dowlatshahi D., MacQueen G. M., Wang J. F. & Young L. T. Increased hippocampal BDNF immunoreactivity in subjects treated with antidepressant medication. Biological psychiatry 50, 260–265 (2001). 11522260 10.1016/s0006-3223(01)01083-6

[b23] Karege F. . Low brain-derived neurotrophic factor (BDNF) levels in serum of depressed patients probably results from lowered platelet BDNF release unrelated to platelet reactivity. Biological psychiatry 57, 1068–1072, doi: 10.1016/j.biopsych.2005.01.008 (2005). 15860348

[b24] Tang J. . Involvement of normalized NMDA receptor and mTOR-related signaling in rapid antidepressant effects of Yueju and ketamine on chronically stressed mice. Scientific reports 5, 13573, doi: 10.1038/srep13573 (2015). 26315757 PMC4551989

[b25] Freitas A. E. . Fluoxetine modulates hippocampal cell signaling pathways implicated in neuroplasticity in olfactory bulbectomized mice. Behavioural brain research 237, 176–184, doi: 10.1016/j.bbr.2012.09.035 (2013). 23018126

[b26] Aguiar A. S. Jr. . Short bouts of mild-intensity physical exercise improve spatial learning and memory in aging rats: involvement of hippocampal plasticity via AKT, CREB and BDNF signaling. Mech Ageing Dev 132, 560–567, doi: 10.1016/j.mad.2011.09.005 (2011). 21983475

[b27] Wang P. . Impaired spatial learning related with decreased expression of calcium/calmodulin-dependent protein kinase IIalpha and cAMP-response element binding protein in the pentylenetetrazol-kindled rats. Brain Res 1238, 108–117, doi: 10.1016/j.brainres.2008.07.103 (2008). 18710651

[b28] Patil S. S., Schlick F., Hoger H. & Lubec G. Involvement of individual hippocampal signaling protein levels in spatial memory formation is strain-dependent. Amino Acids 39, 75–87, doi: 10.1007/s00726-009-0379-8 (2010). 19890699

[b29] Sung J. Y. . Learning strategy selection in the water maze and hippocampal CREB phosphorylation differ in two inbred strains of mice. Learn Mem 15, 183–188, doi: 10.1101/lm.783108 (2008). 18353993 PMC2327260

[b30] Nibuya M., Nestler E. J. & Duman R. S. Chronic antidepressant administration increases the expression of cAMP response element binding protein (CREB) in rat hippocampus. The Journal of neuroscience : the official journal of the Society for Neuroscience 16, 2365–2372 (1996). 8601816 10.1523/JNEUROSCI.16-07-02365.1996 PMC6578518

[b31] Reus G. Z. . Ketamine plus imipramine treatment induces antidepressant-like behavior and increases CREB and BDNF protein levels and PKA and PKC phosphorylation in rat brain. Behavioural brain research 221, 166–171, doi: 10.1016/j.bbr.2011.02.024 (2011). 21397634

[b32] Czeh B. & Simon M. [Neuroplasticity and depression]. Psychiatr Hung 20, 4–17 (2005). 16389729

[b33] Rakhit S., Clark C. J., O’Shaughnessy, C. T. & Morris B. J. N-methyl-D-aspartate and brain-derived neurotrophic factor induce distinct profiles of extracellular signal-regulated kinase, mitogen- and stress-activated kinase, and ribosomal s6 kinase phosphorylation in cortical neurons. Mol Pharmacol 67, 1158–1165, doi: 10.1124/mol.104.005447 (2005). 15625280

[b34] Nakajima S. . Self-amplified BDNF transcription is a regulatory system for synaptic maturation in cultured cortical neurons. Neurochemistry international 91, 55–61, doi: 10.1016/j.neuint.2015.10.009 (2015). 26596846

[b35] Zhang W. V. . [A new way for inbred strain mice genetic monitoring and the discovery of sex-linkaging RAPD markers]. Shi Yan Sheng Wu Xue Bao 29, 59–69 (1996). 9208643

[b36] Shang H., Wei H., Yue B., Xu P. & Huang H. Microsatellite analysis in two populations of Kunming mice. Lab Anim 43, 34–40, doi: 10.1258/la.2008.008098 (2009). 19141464

[b37] McHenry J., Carrier N., Hull E. & Kabbaj M. Sex differences in anxiety and depression: role of testosterone. Frontiers in neuroendocrinology 35, 42–57, doi: 10.1016/j.yfrne.2013.09.001 (2014). 24076484 PMC3946856

[b38] Carrier N., Wang X., Sun L. & Lu X. Y. Sex-Specific and Estrous Cycle-Dependent Antidepressant-Like Effects and Hippocampal Akt Signaling of Leptin. Endocrinology 156, 3695–3705, doi: 10.1210/EN.2015-1029 (2015). 26181103 PMC4588814

[b39] Steru L. . The automated Tail Suspension Test: a computerized device which differentiates psychotropic drugs. Progress in neuro-psychopharmacology & biological psychiatry 11, 659–671 (1987). 2894041 10.1016/0278-5846(87)90002-9

